# Target motion misjudgments reflect a misperception of the background; revealed using continuous psychophysics

**DOI:** 10.1177/20416695231214439

**Published:** 2023-12-21

**Authors:** Michael Falconbridge, Robert L. Stamps, Mark Edwards, David R. Badcock

**Affiliations:** School of Psychology, University of Western Australia, Crawley, Western Australia, Australia; Department of Physics and Astronomy, 8664 University of Manitoba, Winnipeg, Manitoba, Canada; Research School of Psychology, 2219 Australian National University, Canberra, Australia; School of Psychology, University of Western Australia, Crawley, Western Australia, Australia

**Keywords:** higher-order motion, optic flow, perception, perception/action, models

## Abstract

Determining the velocities of target objects as we navigate complex environments is made more difficult by the fact that our own motion adds systematic motion signals to the visual scene. The flow-parsing hypothesis asserts that the background motion is subtracted from visual scenes in such cases as a way for the visual system to determine target motions relative to the scene. Here, we address the question of why backgrounds are only *partially* subtracted in lab settings. At the same time, we probe a much-neglected aspect of scene perception in flow-parsing studies, that is, the perception of the background itself. Here, we present results from three experienced psychophysical participants and one inexperienced participant who took part in three continuous psychophysics experiments. We show that, when the background optic flow pattern is composed of local elements whose motions are congruent with the global optic flow pattern, the incompleteness of the background subtraction can be entirely accounted for by a misperception of the background. When the local velocities comprising the background are randomly dispersed around the average global velocity, an additional factor is needed to explain the subtraction incompleteness. We show that a model where background perception is a result of the brain attempting to infer scene motion due to self-motion can account for these results.

## Background

**Figure 1.**
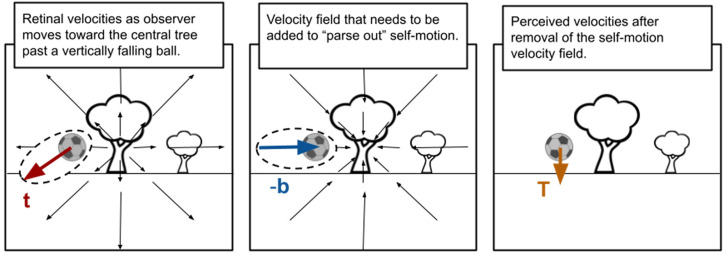
Depiction of the flow-parsing hypothesis. In order to calculate the actual (world-centered) motions of the objects in a scene (e.g., the soccer ball velocity, **T**, depicted in the right panel) when traveling through the scene, the motion signals due to self-motion need to be subtracted (e.g., –**b**, middle panel) from the motion signals experienced by the observer (e.g., **t**, left panel). The flow-parsing hypothesis asserts that the visual system subtracts unattended motion signals from target motion signals in this manner in order to obtain world-centered perceived target motions.

During everyday activities such as driving through a busy intersection and catching a ball on the run, it is important that the visual system accurately calculates the motions of objects in the scene that are relevant to the task at hand. In both of these examples, the observer is in motion and their self-motion adds systematic velocity signals to the scene making the task of extracting the motion of attended objects potentially more difficult. The flow-parsing hypothesis proposes that, under such conditions, the visual system determines what motion signals are due to self-motion and subtracts them from the object-motion signals to get object-motions relative to the scene, rather than the self. In other words, the observer perceives *allocentric* or world-centered object motions (Rushton & Warren, 2005; Warren & Rushton, 2007). See Figure 1 for a depiction of this process. This hypothesis has received overwhelming support in recent years (e.g., Dupin & Wexler, 2013; Fajen et al., 2013; Falconbridge et al., 2022b; Foulkes et al., 2013; Mayer et al., 2021; Rogers et al., 2017; Warren & Rushton, 2008; Warren & Rushton, 2009) and provides an explanation for the misperception of target motion in the classic Induced Motion Illusion (Falconbridge et al., 2022b; Rushton & Warren, 2005; Warren & Rushton, 2004).

The process depicted in Figure 1 can be described using vector notation. The perceived target motion **T** (depicted in the right panel of Figure 1), is calculated as

(1) T=t–b

where **t** is the motion of the target in the scene (left panel) and **b** is the motion of the background in the vicinity of the target (also in the left panel). Note that Figure 1 and equation (1) represent an ideal. It has been shown repeatedly in lab settings where this subtraction process has been studied that the subtraction is rarely complete. In practice

(2) T=t–βb

where β < 1 (reviewed in Mayer et al., 2021). β is commonly referred to as the “flow-parsing gain” in the literature (Niehorster & Li, 2017) and tends to vary between about 0.5 and 0.8 when only visual stimuli are used (Dokka et al., 2013; Dokka et al., 2015; Dupin & Wexler, 2013; Falconbridge et al., 2022b; Mayer et al., 2021; Xie et al., 2020) and has been shown to reach as high as 0.98 when congruent vestibular and other inputs are combined with the visual stimulus (Xie et al., 2020).

## Experimental Questions

In this study, we are interested in two questions related to β. Both questions have received little attention in the literature: what mechanism underlies the subtraction incompleteness in lab settings and how is the background motion perceived in scenes such as those depicted in Figure 1? We wondered, in particular, whether the two questions might be related, that is, is the incompleteness of the background subtraction due to a miscalculation of the background motion by the visual system, or is it due to a shortcoming in the subtraction process itself? If it were due to a miscalculation of the background motion then, we surmised, there should be an associated mis*perception* of the background.

**Figure 2.**
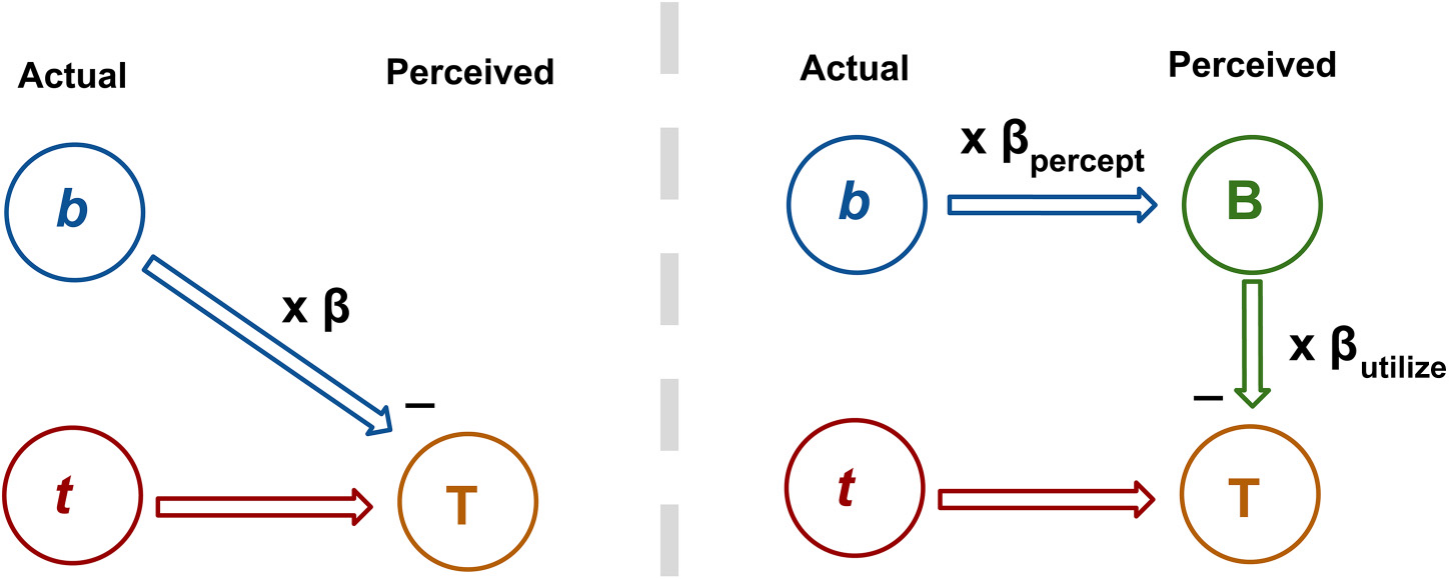
Bisection of the background pathway by probing the perception of the background **B**. On the left is a depiction of the vector subtraction process (equation (2)). By probing perceived background motion, the **b**-to-**T** pathway is split into two parts which (potentially) divides β into two components—that which is applied pre-perception (β_percept_) and that which is applied afterward (β_utliise_).

The way our two questions relate to one another can be depicted simply in the following way: there is a process within the visual system that links the stimulus background motion **b** to perceived target motion **T** and somewhere within that process β is applied (see left panel of Figure 2). In this study, we inserted a psychophysical probe along with the background processing stream by looking at perceived background motion **B**. This is depicted in the right panel of Figure 2.

By examining perceived background motion **B**, one can break β into two parts—that which is applied prior to the stage associated with background perception and that which is applied afterward. These β values will be referred to as β_percept_ and β_utilize_, respectively. The subscript “utilize” indicates that the second β value determines how much the perceived background motion is used in the subtraction process depicted by the “-” near **T**. Note that multiplication by β has been replaced with two multiplication steps making β_total_ = β_percept_ × β_utilize_.

By probing a participant's perception of the background motion we aim to (1) localize β (<1) to a position in the background processing pathway—before the area associated with background perception, after that area or a component of β before and a component after—and (2) gain a sense of how participants perceive the motion of the background. This will allow us to see if a misperception of the background (β_percept_ ≠ 1) is responsible for β_total_ being less than one.

Note that we cannot be *sure* that perceived background motion **B** is associated with a visual processing area between **b** and **T** as depicted in Figure 2 (resulting in a bisection of the background pathway), but we hold that as an assumption at this stage in the study and in the General Discussion consider whether the data presented here are supportive of that assumption.

In order to answer our two questions we took the following two steps: (1) we measured β_total_ under a range of conditions and (2) we measured β_percept_ under those same conditions so that β_total_ could be parsed into its two components (β_percept_ and β_utilize_). The three conditions under which β_total_ and β_percept_ were measured are distinguished by background motion type: one background was composed of uniform horizontal translation, one was composed of radially expansive flow as in Figure 1, and the last background was composed of random local motions with a global average horizontal translation. The reasons for these particular choices are outlined in the introductory sections for each experiment.

Note that step 1—the measuring of β_total_ under a range of conditions—has been performed extensively in a range of Induced Motion and Flow-Parsing studies (Dokka et al., 2013; Dokka et al., 2015; Falconbridge et al., 2022b; Mayer et al., 2021; Niehorster & Li, 2017; Xie et al., 2020). Our study is distinguished from these previous studies by the fact that we measured β_total_ using a *continuous* psychophysics approach described below. We argue that our continuous approach allows for the study of this phenomenon under more natural conditions and offers other advantages over traditional trial–based approaches. We could find only one study that performed step 2. Rock et al. (1980) used a moving rectangular frame as a “background” and a spot of light as a “target” and asked participants to judge the motion of the frame (as well as the spot) thus directly quantifying background perception. Again, our study is distinguished by our use of a continuous psychophysics approach.

Having presented the two questions we wish to address in this study and having outlined the steps we took to answer them, we describe the continuous regime employed in our experiments as this regime was key in allowing us to take these steps in a reasonable amount of time and was important for a number of other reasons outlined below. Below we also briefly outline the unique method we needed to employ to analyze the continuous data.

### A Continuous Approach

Our continuous psychophysics paradigm was previously developed in our lab (Falconbridge et al., Under Review). In this paradigm, participants experience a constantly varying stimulus and are tasked with responding to the changing stimulus continuously over an extended period of time. Each continuous session in our current study lasted just over 4 min. To collect the equivalent amount of data to that in a 4-min session using trial-based methods took about 3 h in a study conducted previously in our lab (Falconbridge et al., Under Review). Thus, the continuous approach offers significant savings in participation time. Collecting data *quickly* allowed us to test various conditions that might affect β_percept_ and β_utilize_ for each participant in a reasonable amount of time. As well as offering a means of rapid data collection, four more considerations make a continuous approach desirable in a flow-parsing study:

1. Accurate heading perception, which is essential for knowing what parts of the motion field are due to self-motion (Foulkes et al., 2013), requires exposure to simulated self-motion scenes that vary with time (Burlingham & Heeger, 2020). The unchanging or “instantaneous” optic flow stimuli that are used in typical trial-based experiments are, thus, impoverished when it comes to the study of flow-parsing.
2. One thing that has been shown to affect flow-parsing gain is multisensory stimulation (e.g., Mayer et al., 2021; Xie et al., 2020). In particular, the addition of vestibular cues that are conducive with the background motion has been shown to increase β_total_. It is difficult and time-consuming to add natural-feeling and appropriate vestibular cues on a trial-by-trial basis. A continuous approach allows for the seamless addition of physical motion that is either congruent or not with the continuously varying background motions. In the General Discussion, we discuss an experiment conducted previously in our lab where such physical motion was employed.
3. A continuous method uses stimuli that vary continuously with time and engages natural perception-action loops. This mimics situations that the flow-parsing mechanism evolved to deal with. Using a continuous approach one can test whether the results obtained using more abstract, trial-based approaches hold under more natural conditions.
4. A positive side-effect of this naturalistic interaction with the stimulus is that the participant's task is engaging and requires sustained attention which alleviates boredom effects (Huk et al., 2018).

The component of the scene that was adjusted continuously in all experiments was the background motion **b**. The component that was adjusted by the participant was the target motion **t**. Our continuous method, thus, allowed us to collect **t** vectors for a *large range* of **b** vectors which, then, allowed us to discern the nature of the relationship between **t** and **b**. For example, by making certain assumptions about the perceived target direction, **T**, we were able to confirm that a simple subtraction was being performed by the visual system, that is, that **T** = **t** – β**b** as assumed by the flow-parsing hypothesis. At the same time, we were able to determine values for β; both β_percept_ and β_total_.

Recall that step 1 for answering our experimental questions was to measure β_total_ under a range of background conditions. Measuring β_total_ equates to measuring the extent to which the background motion vector in the vicinity of the target **b** is subtracted from the target motion **t** to produce perceived target motion **T** (see equation (2)). This was measured in our study using a continuous *correction* task. For a given background motion **b**, we had participants choose a target motion **t** that produced vertical perceived target motion **T**. We continuously perturbed the background motion so that participants were forced to make continuous “corrections” to the target motion so that it always appeared as close to vertical as possible.

In relation to step 2, measuring β_percept_ equates to measuring the extent to which *perceived* background motion in the vicinity of the target **B** matches the *actual* background motion in that part of the scene **b**. This was measured using a continuous *matching* task. To obtain perceived background motion in the vicinity of the target for a given background motion, we had participants match the target motion to that of the perceived background in the vicinity of the target. As with the continuous correction task, the actual background motion was continuously perturbed, thereby forcing participants to make continuous adjustments to the target motion.

In order to analyze the inherently noisier data produced using our continuous approach, a Bayes-optimal model of the participant was employed. Here, both the sensory and action components of the model participant perform in a Bayes-optimal manner (Falconbridge et al., Under Review). Using this model, we traced the stimulus via the sensory stream and the actions via the action stream to the “decision center” of the model which gave us a sense of the stimulus properties to which the participant was ideally responding and the ideal actions they planned as a result. We have shown previously that these ideal stimuli and responses are comparable to actual stimuli and responses in much more controlled trial-based experiments (Falconbridge et al., Under Review). More detail about our analysis method is offered under the Methods section of Experiment 1 below.

In summary, the continuous approach combined with our analysis method offers reliable data, similar to that obtained from trial-based experiments, but in a fraction of the time with the added benefit of employing engaging and naturalistic data-collection sessions. These sessions more closely simulate environments under which the visual system evolved its target-background parsing mechanism and the continually changing stimulus allows for more accurate perception of heading direction.

## Experiment 1: Induced Motion

In this experiment, we estimated both β_percept_ and β_total_ using a continuous version of a stimulus similar to that typically used in Induced Motion experiments (e.g., Zivotofsky, 2004). The reason for using this stimulus is that it connects our study to the large body of literature on the Induced Motion effect and it represents an uncomplicated background—one where all local elements move at the same speed and direction. The background motion consisted of uniform horizontal translation but the magnitude and sign of that motion varied with time so as to perform a random walk. At each time step (duration 0.1 s), a left or right facing low-magnitude motion vector was added to the current motion of the background with a 50% chance of each.

### Methods

#### Participants

All but RS were experienced, psychophysical observers. All but ED were aware of the purposes of the study but only MF was aware of how the experimental tasks related to that purpose. Participant ED had a divergent squint and completed the experiments using an opaque eye patch over the nondominant eye. All participants gave their informed consent to the study which had ethics approval (RA/4/1/4503) from the Human Ethics Committee at the University of Western Australia and therefore conformed to the tenets of the Declaration of Helsinki.

#### Apparatus

Stimuli were displayed on a SONY Trinitron G420 monitor (1024 × 768 pixels at 100 Hz) placed 57 cm from a chin rest. Stimuli were generated and updated using Unity version 2018.4.7f1 running on a pc with a Windows 10 operating system. The target direction or speed was adjusted by participants using left-right motion of a mouse.

#### Stimuli

We used the same flexible visual stimulus across all experiments. In order to have target and background motion without either stimulus component changing their position relative to fixation and without them leaving/entering the display area during a continuous session we used “dynamic global plaid” stimuli (c.f. Global Gabor Stimuli in Amano et al., 2009) where the target and background were each made up of small plaid patches where each Gaussian “container” remained in place but the plaid patterns within the containers drifted with time. Target plaid patterns drifted in the global direction and at the global speed of the target, and background patterns drifted at the local velocity that was congruent with the optic flow of the background. It has been shown previously that the subtraction (of background from target velocity) effect, thus produced, is the same as that obtained when using more traditional moving stimuli like fields of dots (Falconbridge et al., 2022b). This stimulus is capable of representing a range of optic-flow types without requiring a change to the layout or composition of the scene.

In Experiment 1, the background motion was uniform sideways translation. See Video 1 for a movie of the Experiment 1 stimulus.

The sideways background motion shown in this video simulates one of two real-world scenarios: sideways translation of the participant where all background elements lie on a plane at a certain distance from the participant or rotation of the participant with no restrictions on the distance of background elements from the participant. Varying the background motion continuously simulates continuously changing the sideways motion or rotation of the participant. Note that the background motion just described is a continuously varying version of backgrounds used in most traditional induced motion experiments.

The target consisted of 30 Plaid patches evenly distributed around a 4° radius ring centered on the display. The background consisted of 40 Gabor elements randomly scattered over a 20° by 20° region centered on the display (elements were free to also appear inside the target ring). Plaids were composed of a pair of orthogonal 3 cycle/degree orthogonal gratings, each with a Michelson contrast of 0.40, within an 8′ Gaussian window. Orientations for both the target and background were randomized at the beginning of each data-collecting session. The background direction was always horizontal but the speed varied with time, stepping more leftward or rightward by a certain amount every 0.1 s with a 50% chance of each. The step size was exactly equal to the horizontal component of a 1° change in direction for a virtual “background” that moved at the same speed as the target and whose horizontal component of motion was equal to the horizontal speed of the actual background. This meant bigger step sizes when speeds were low and smaller ones when speeds were higher. This was to compensate for the fact that a larger change in direction for the target would be needed for the same change in background speed when background speeds were higher. The reason for this is that the target direction would be further away from vertical so a given change in target direction would produce a smaller change in the horizontal component of motion. For the continuous correction task, used to determine β_total_, the target plaids “drifted” at 2.5 °/s at all times with the direction of drift being controlled by the participant. A right movement of the mouse caused a clockwise change in direction and a left movement caused an anticlockwise change. This process is referred to as target “steering” in what follows. For the continuous matching task, used to determine β_percept_, the target direction of motion was horizontal, and the speed was controlled by the participant. A leftward mouse movement caused an increase in leftward speed and rightward movement an increase in rightward speed.

#### Procedure

The Bayesian Participant model was used to analyze experimental data. In order to fit the model parameters to each participant, participants first took part in a training session. In this session, they practiced using left-right movements of the mouse to steer the target toward a 2° Gaussian luminance blob that moved around the outside of the display area along an arc with a radius of 12° centered on fixation. This blob stepped 10° either clockwise or anticlockwise of its current position, with a 50% chance of either, every 5 s. The steering responses of participants to this stepping blob were used to estimate damped spring constants and noise variances needed to fit the parameters of the Bayesian Participant model to each participant for future data analysis. Each background plaid in the display drifted in a random direction and at a random speed (up to 2.5 °/s) set at the beginning of the training session to get participants used to movement in the background. Training sessions lasted 2 min and 10 s where the first 10 s were not used. See Falconbridge et al. (Under Review) for more detail on training sessions.

Participants then took part in three continuous correction sessions and three continuous matching sessions. Each session lasted 4 min and 10 s where the first 10 s were discarded from analysis thereby allowing time for participants to get a “feel” for the scene and for the control of the target prior to data collection. In the continuous correction sessions, the participants were tasked with keeping the target moving vertically upward. This required correcting for any perceived deviations from vertical motion whenever this was perceived. In the continuous matching sessions, they were tasked with matching the sideways motion of the target with the perceived global sideways motion of the background. For both types of sessions, so that the background sideways speed could undergo a true random walk, random walks were simulated offline prior to each session until a walk was found that covered the desired speed range—from 2.5 °/s leftward to 2.5 °/s rightward with a buffer of 0.15 °/s either side.

Participants were free to take part in as many practice sessions for each task/condition as they needed to get comfortable with the task before completing data-collection sessions. This is the case for all conditions that follow. The number of practice sessions tended to lie between one and five 4-min sessions.

#### Data Analysis

Training sessions were analyzed using the BP_training_analysis.m Matlab script in the Supplemental section. This script takes the responses to each blob step and uses the average response to estimate how long it takes for a participant to begin a response to a stimulus change, how much noise there is in their internal representation of stimulus direction, and how “springy” and “damped” their movements are. These estimates are used in the analysis of data for all following sessions.

Continuous correction sessions were analyzed using the BP_correction_analysis.m Matlab script in the Supplemental section. This script converts the target motions set by the participant, **t**, and the background motions, **b**, into “ideal” versions of these motions. These ideal versions are Bayes-optimal estimates of **t** and **b** obtained by a model participant whose “retina” adds Gaussian noise to **t** and **b** in the transduction process and whose “brain” hosts an accurate model of the external stimulus generative process. The ideal estimate of **t** also accounts for the fact that sometimes perceived target motion is not vertical. It is, in fact, the deviations from verticality that drive the actions of the participant. A model of the action system of the participant is used to estimate when target perceptions are not vertical and by how much. It was shown in Falconbridge et al. (Under Review) that the “ideal” versions of **t** and **b** calculated in this way are similar to **t** and **b** data obtained using more traditional trial-based approaches. Note that there was no evidence of an adaptation effect like that described in Falconbridge et al. (Under Review) so no effort was made to correct for adaptation.

Continuous matching sessions were analyzed using the BP_matching_analysis.m Matlab script in the Supplemental section. This uses the Bayesian Participant model in the same way the continuous correction analysis program does. It does so because the matching task involves the same underlying mathematics as the correction task. For the matching task, we created a new variable **T_match_** which represents the target motion relative to the perceived background motion, that is, **T_match_** = **t** – **B**, which is just **t** – β_percept_**b** according to our model. The aim in the matching task is to maintain **T_match_** at zero as well as possible. This is exactly equivalent to the equation underlying the continuous correction task **T** = **t** – β_total_**b** (equation (2)). As vertical is defined as direction 0, the aim of the participant in the correction task is to make **T** = 0 just as the aim is to make **T_match_** = 0 in the matching task.

As the background motion is horizontal only, we looked only at the horizontal components of **b**, **t**, and **T**.

### Results

**Figure 3.**
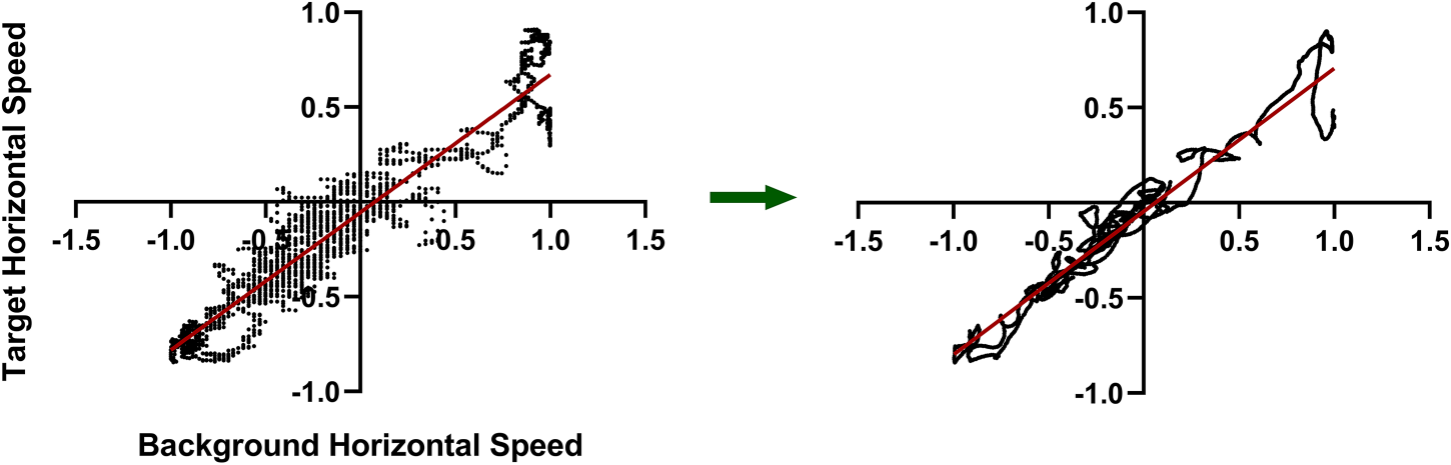
Raw and processed data. Plotted on the left are the horizontal components of target and background velocities sampled 20 times per second during a 4-min continuous correction session for participant RS. On the right is the same data after being processed with the use of our Bayesian Participant model. In red is shown the line of best fit. The slope represents β as, assuming perceived target direction is straight up, then, for the horizontal components, **T **= **t** – β**b** becomes 0 = t_x_ – βb_x_ i.e., t_x_ = βb_x_. Note that the spread in the data is reduced and the consistency of the data with the model is increased by processing (see text for details). Axes represent speeds in native Unity units where 1 equates to 2.5 deg/s.

In the left panel of Figure 3, typical raw data are shown from one of the 4-min data-collection sessions. On the right is the processed data following the application of our Bayesian Participant model. The best-fit line where the slope represents β is shown in red. The slope specifically represents β_total_ as these data comes from a continuous correction session.

Consistent with previous work in our lab (Falconbridge et al., Under Review), the relationship between the target and background is clearer in the processed data; there is less variation in the data and the data are more consistent with the (linear) model. For example in Figure 3, R^2^ increased from 0.908 to 0.943 and the standard deviation of the residuals (Sy.x) fell from 0.137 to 0.108. For the group, average R^2^ rose significantly from 0.934 to 0.957 (*t*[11] = 6.28, *p* = 0.000006, paired two-tailed *t*-test) and Sy.x fell significantly from 0.127 to 0.097 (*t*[11] = 7.88, *p* = 0.0000008, paired two-tailed *t*-test) for the continuous correction data. This pattern was consistent throughout this study for both the continuous correction and the continuous matching tasks.

Reduction in noise aside, the β values themselves were not significantly altered by the application of our Bayesian Participant analysis method.

There were no discernible systematic patterns in the residual plots indicating that a simple linear model of the form **T **= **t** – β**b** was sufficient for describing both the matching and correction results. This, in turn, indicates that the visual system was indeed applying a function that globally equates to a simple subtraction. This was the case for all three experiments.

**Figure 4.**
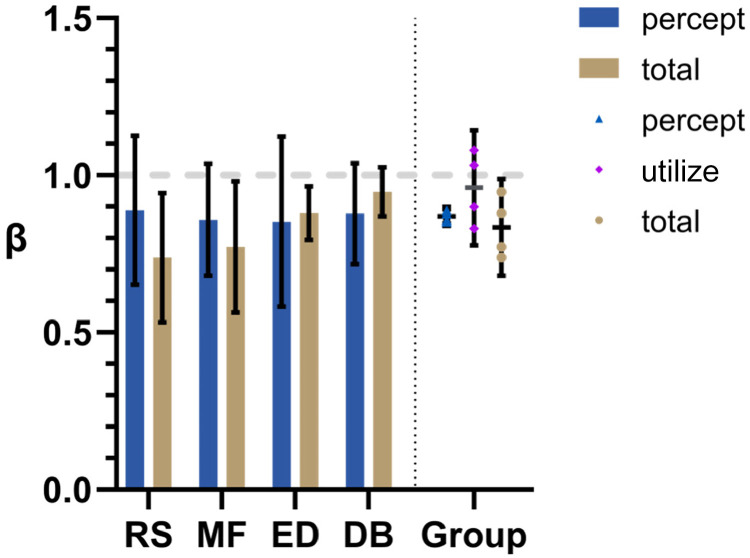
Induced motion results. On the left are bar graphs depicting mean β values for both the matching (“percept”) and correction (“total”) tasks under the Induced Motion condition. Here, the background motion was laminar translational flow as in standard Induced Motion experiments. On the right is a summary of the group means for both β_percept_ and β_total_ plus calculated β_utilse_ values. Error bars represent 95% CI.

Mean β_percept_ and β_total_ values are plotted for each participant on the left side of Figure 4. On the right are shown group means for β_percept_ and β_total_ along with calculated β_utilize_ values. The same four participants were used in Experiments 1–3 so that comparisons between conditions could be easily made. Error bars represent 95% confidence intervals.

As expected from previous studies, β_total_ values were less than 1; significantly for RS (*t*[2] = 5.50, *p* = 0.03, two-tailed *t*-test), MF (*t*[2] = 4.70, *p* = 0.04, two-tailed *t*-test), ED (*t*[2] = 6.10, *p* = 0.03, two-tailed *t*-test), and the group as a whole (*t*[3] = 3.43, *p* = 0.04, two-tailed *t*-test). Perhaps surprisingly, the mean β_pecept_ is also significantly less than 1 for the group (*t*[3] = 15.62, *p* = 0.00006, two-tailed *t*-test). Mean β_percept_ is also very close to mean β_total_ for the group making mean β_utilize_ close to 1 (not significantly different to 1; *t*[3] = 0.69, *p* = 0.54, two-tailed *t*-test).

Included in the Supplementary section is a video (Video 4) of the target and background while MF performed the matching task used to calculate β_pecept_. While focussing on the target it may appear that its motion matches, on average, that of the background with deviations occurring when the background motion randomly changes in a significant way. But if the video is sped up, either forward or backward by sliding the bar on the timeline, it becomes clear that the target is moving at a slower rate than the background, thus β_pecept_ < 1.

Our participants all reported that during both the matching and correction tasks, the background motion was only rarely noticeable. Some readers may experience this as they focus on the target in Video 4. This is consistent with many Induced Motion studies where a moving background appears stationary (and the stationary target appears to be moving) (Reinhardt-Rutland, 1988). As the aim of the participant in Video 4 was to match the target to the background some readers may experience a complete absence of motion, both in the target and the background.

### Discussion

The matching results may be surprising. In this experimental condition, the motion of the background was uniform across the entire display and it was clear (no noise). Even so, participants failed to match the target to the *actual* motion of the background. Instead, the target was set, on average, to about 0.88 of the background speed and this was consistent across participants, that is, β_pecept_ was about 0.88 on average. This average was not significantly different from that for β_total_. That is, there is no evidence to suggest that the misperception of the target (β_total_) was not entirely due to a misperception of the background (β_pecept_).

## Experiment 2: Expansive Optic Flow

**Figure 5.**
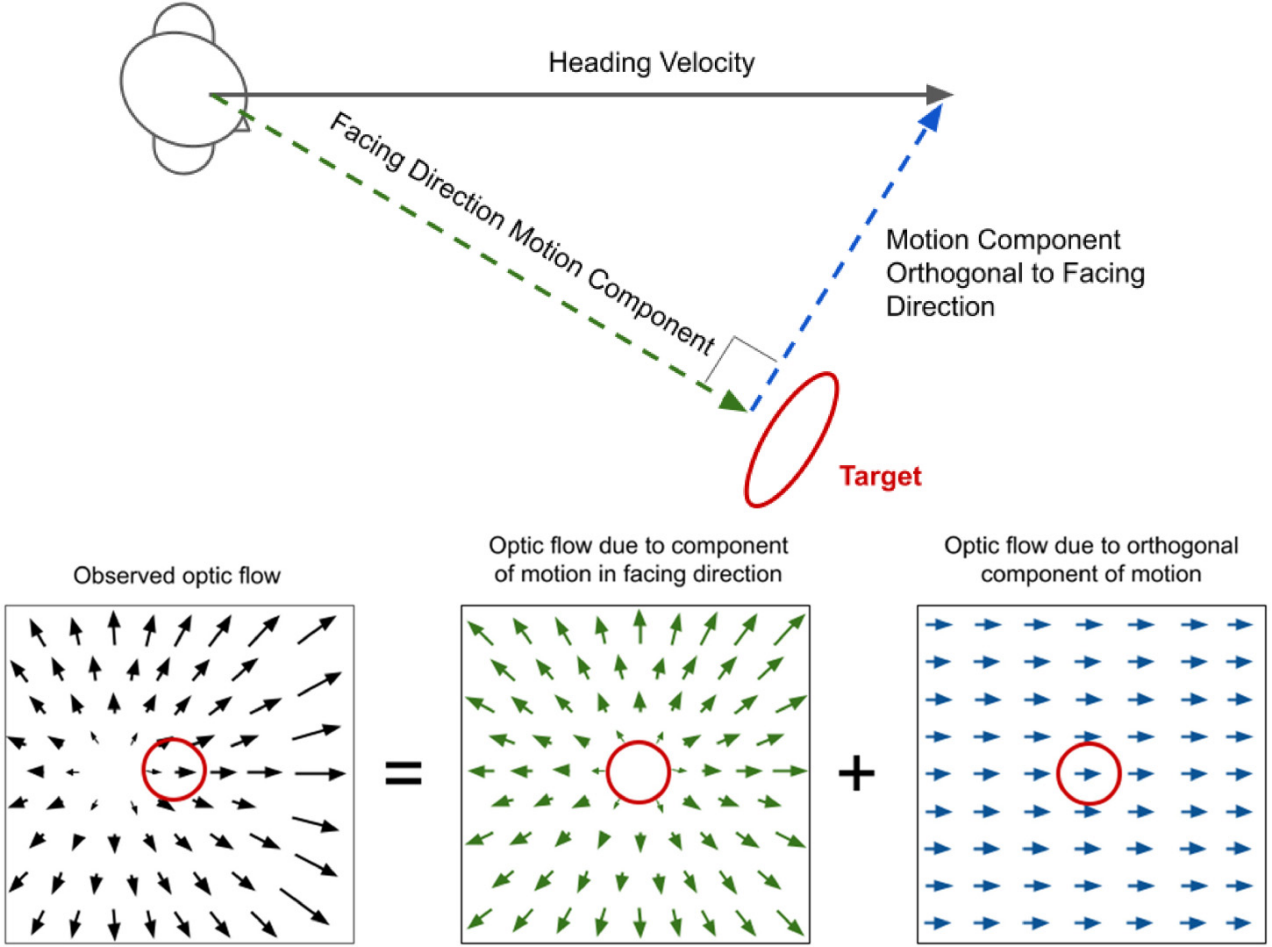
Optic flow when fixation is offset from heading direction. The top image depicts a person facing a red circular target that is to the right of where they are traveling. The actual velocity of the person (their heading velocity) can be decomposed into two components—one in the direction they are facing, and the other orthogonal to that direction. The bottom panels show that the resulting optic flow pattern can be decomposed into two parts corresponding to self-motion in the two-component directions. It is only the “sideways” optic flow component corresponding to motion in the direction orthogonal to the facing direction (right panel) that affects perceived target motion if optic flow is subtracted from the target motion (as motion in the central panel is symmetric near the target). Note that this motion is the same as that at the center of the target in the leftmost panel. A visual system capable of correctly using optic flow information in the subtraction process will be able to extract the sideways component depicted in the right panel and use it in the subtraction process.

Experiment 2 stimuli were composed of local elements whose speeds were proportional to distance from the Focus of Expansion (FOE) and whose directions were along with radii emanating from the FOE similar to the motion depicted in Figure 1. See Video 2 for an example video. This represents an incremental increase in background complexity compared to Experiment 1 and it links our study to a bulk of the flow-parsing literature where this type of flow is commonly employed. Linear expansive optic flow simulates forward motion of the participant toward a plane. The FOE was randomly perturbed horizontally in a manner similar to the horizontal velocity in Experiment 1—stepping left or right by a certain amount every 0.1 s. Having the FOE off to one side of fixation represents the participant directing their gaze to one side of the direction they are heading in. This is depicted in Figure 5. Fixing one's gaze to one side of the heading direction introduces a sideways motion to the background. This sideways motion is equivalent to the sideways motion in Experiment 1 already described and is the only part of the background motion that should be used in the **T **= **t** – β**b** subtraction process. Just as directly varying the sideways velocity in Experiment 1 alters **b**, so does varying the sideways position of the FOE in Experiment 2.

As in Experiment 1, we measured β_percept_ and β_total_. To measure β_percept_, participants attempted to match the target to the sideways motion in the display in the vicinity of the target which is equivalent to the sideways motion depicted in the right panel of Figure 5. To measure β_total_ they attempted to keep the perceived target moving vertically upward. These are the continuous matching and continuous correction tasks, respectively.

### Method

#### Participants

The participants were the same as Experiment 1.

#### Stimuli

The target stimulus and the control of the target motion by the participant was the same as in Experiment 1. What differed was background motion. Here, the horizontal position of a point representing the FOE underwent a random walk just as the sideways speed of the background underwent a random walk in Experiment 1. Step size was 0.15° with a 50% chance of a leftward or rightward step every 0.1 s. The drift of each plaid patch was determined by its relationship to the FOE; speed increased proportionally with distance from the FOE at a rate of 0.5 °/s per degree and direction was always radially away from the FOE. See Video 2 in the Supplementary section.

#### Procedure

Participants took part in three continuous correction sessions and three continuous matching sessions. The continuous correction and continuous matching tasks were the same as in Experiment 1. Participants kept the target moving upward in the correction sessions and matched the target to any perceived global sideways motion in the background during matching sessions.

Random walks were chosen prior to each session so that the FOE covered a 12° horizontal range centered on the display, making the maximum global sideways motion 3 °/s.

#### Data Analysis

Continuous correction and continuous matching results were analyzed in the same way as they were for Experiment 1 except that the background sideways motion used as input to the analysis program had to be extrapolated from the position of the FOE. The extrapolation was based on the vector addition mechanism depicted in Figure 5, for example, when the FOE was at its maximum rightward position of 6° from fixation the global sideways motion was 3 °/s leftward. Participants were presented with stimuli like that in the lower left panel of this figure. The sideways motion used as input is represented in the lower right panel and is the motion orthogonal to the simulated fixation direction of the participant.

Note that only the first 3 min of the 4-min session were used for analysis for one of RS's matching sessions because, in the participant's own words, they “lost control of the target” toward the end. This manifests in the data as a large sweeping of target direction across direction space, presumably in an attempt to regain verticality in target perception.

### Results

**Figure 6.**
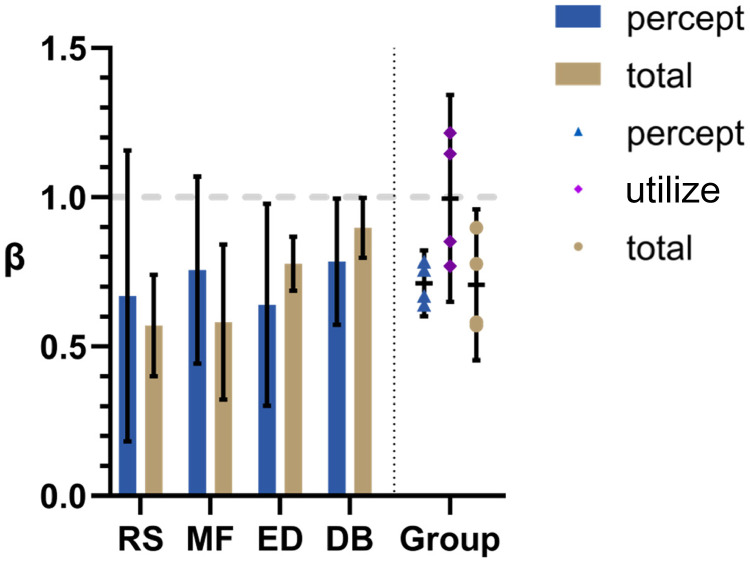
Linear expansive optic flow. Here, participants viewed radial optic flow where the speed varied proportionally with distance from the FOE. Mean β_percept_ and β_total_ values are shown for individuals on the left and group means are shown on the right. Error bars represent 95% CI.

As with Experiment 1, all processed data were well modeled with the linear equation **T **= **t** – β**b** for both the correction and matching tasks, supporting a simple subtraction mechanism. Mean β_percept_ and β_total_ values are shown in Figure 6 for individuals on the left and for the group as a whole on the right.

The pattern of results for the Optic Flow condition was very similar to that in Experiment 1. Both mean β_percept_ and β_total_ values were significantly less than 1 for the group (*t*[3] = 8.33 and 3.69, *p* = 0.004 and 0.03, respectively, two-tailed *t*-test) and both means were very similar. As with Experiment 1, this made mean β_utilize_ almost exactly equal to 1 for the group (not significantly different from 1 with *t*[3] = 0.04, *p* = 0.97, from a two-tailed *t*-test). The only difference between these group results and those in Experiment 1 is that mean β_percept_ and β_total_ values were smaller for this (Optic Flow) condition. This difference was significant (*t*[3] = 4.58 and 4.00, *p* = 0.02 and 0.03 for β_percept_ and β_total_, respectively, from a two-tailed paired *t*-test).

### Discussion

The Experiment 2 results lend further support to the idea that β_total_ (misperception of the target) is entirely due to β_percept_ (misperception of the background).

## Experiment 3: Randomized Flow Field

For the Randomized Flow Field condition, the stimulus generation process was exactly the same as for the Optic Flow condition in Experiment 2 but the random position in which each element appeared did not correspond to the random position used for calculating the motion of that element. This is comparable to Warren & Rushton’s (2008) “vector shuffling” technique. It meant that the distribution of speeds and directions of elements was exactly the same as that in the flow condition but the radial expansive structure was absent—replaced with a field of apparently randomly moving elements. What was common between conditions in a global sense was the 2D *average* motion.

This condition was used for three reasons. Firstly, and most importantly, it was a means of testing how target and background perception is affected by the addition of noise in the background optic flow pattern. In this case, the global optic flow was sideways translation and the noise was in the form of a random spread in the velocities of the local elements about the global velocity. Secondly, it was used to confirm the findings of Warren & Rushton (2008) that background optic flow structure is important to target perception. Does using the same local elements as Experiment 2 but randomizing their positions so that the optic flow structure disappears change the target perception as was found in Warren & Rushton (2008)? We wanted to confirm that this was the case under the continuous conditions used in our study. Thirdly, if there *is* a difference in target perception when optic flow structure is destroyed, we wanted to determine where the difference occurs in the visual processing stream; is it due to a change in the background perception (β_percept_) or a change in the way the background is used to get target perception (β_utilize_)?

### Method

#### Participants

The participants were the same as in Experiments 1 and 2.

#### Stimuli

The stimulus framework and the method of control of the target motion by the participant was the same as in Experiments 1 and 2. All that differed was the background. Here, each plaid patch had two random positions (within the 20° by 20° background area) assigned to it. The first was used to calculate the continuously varying drift rate for the plaid pattern in exactly the same way it was calculated in Experiment 2. The second described the position in which the plaid patch appeared on the display. In this way, the average motions of the local motion elements were exactly the same as that in Experiment 2, but the positions in which those elements appeared were random, replacing the global radially expanding optic flow pattern with a field of apparently random motions. See Video 3 in the Supplementary section.

#### Procedure

Participants took part in three continuous correction sessions and three continuous matching sessions just as in Experiments 1 and 2.

#### Data Analysis

Data were analyzed in exactly the same way as Experiment 2 except that the background sideways motion was taken as the average of the local motions. Note that, because the relationship of speed to distance from the FOE is linear, this is exactly equivalent to working out the sideways motion based on the position of the FOE as was done in Experiment 2.

### Results

**Figure 7.**
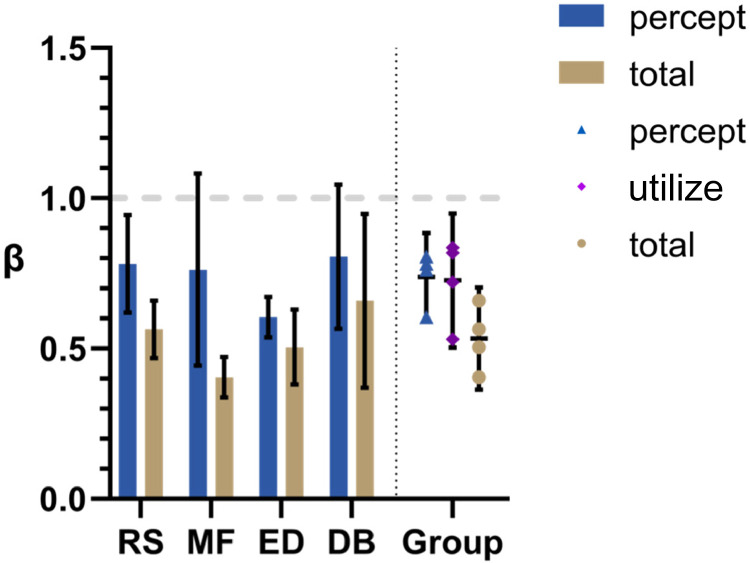
Randomized condition. Here, the stimulus generation process was identical to that in the Optic Flow Condition of Experiment 2 except that local elements did not appear in the position that was used to calculate their velocity, rather they appeared in a randomly assigned position on the display. This meant that the average sideways motion in the background was the same as that in Experiment 2 but the global optic flow pattern was absent; local motions were, instead, “random”. Mean β_percept_ and β_total_ values are shown for individuals on the left and group means are shown on the right. Error bars represent 95% CI.

The pattern of results was *not* the same as that in Experiments 1 and 2 (see Figure 7). Here, the mean β_total_ value was significantly smaller than the mean β_percept_ value for the group (*t*[3] = 3.65, *p* = 0.04, from a two-tailed paired *t*-test). This led to the calculated mean β_utilize_ value being significantly less than 1 (*t*[3] = 3.91, *p* = 0.03, from a two-tailed *t*-test). Note that the different pattern was *not* due to a significant difference in β_percept_ (*t*[3] = 0.84, *p* = 0.46, from two-tailed paired *t*-test), but to a decrease in β_total_ (*t*[3] = 2.94, *p* = 0.03, from a one-tailed paired *t*-test).

### Discussion

The finding that the pattern of results was different for this condition compared with the Optic Flow condition supports the notion that it was not simply the average of the local 2D motions in the optic flow pattern that was used in the subtraction process under the Optic Flow condition in Experiment 2. Average sideways flow was the same in both conditions, what varied was the absence of optic flow structure in Experiment 3. This indicates that optic flow structure is important to target perception under our continuous psychophysics conditions just as it is under trial-based conditions (Warren & Rushton, 2008). The difference between Experiment 2 and Experiment 3 appears to be due, not to a difference in the perception of motion in the background (β_percept_), but to a difference in how that background motion is *used* in the subtraction process (β_utilize_). This point is taken up in General Discussion.

Speaking to our primary drive behind Experiment 3, our results also suggest that when noise is added to the optic flow pattern in the form of a spread in the local velocities around the global mean, target misperception (β_total_) cannot be entirely put down to a misperception of the background (β_percept_). Here, the extent to which the misperceived background is used in the subtraction of background motion from target motion (β_utilize_) also plays a role, that is, β_utilize_ is significantly less than 1.

Another pattern in the data that suggest that β_utilize_ plays an important role in target perception is the fact that, despite the group average β_utilize_ being close to 1 for both Experiment 1 and Experiment 2, it varied systematically with participants across all three experiments; β_utilize_ was smallest for RS and MF and largest for ED and DB. This participant effect was significant (*F*_(3, 6)_ = 9.83, *p* = 0.01, a two-way ANOVA). This is discussed further in General Discussion.

## General Discussion

Our results support those cited in the Introduction showing that, in lab settings, the subtraction of background motion from target motion is incomplete, that is, β_total_ tends to be less than 1 (Dokka et al., 2013; Dokka et al., 2015; Falconbridge et al., 2022b; Mayer et al., 2021; Niehorster & Li, 2017; Xie et al., 2020). Whether this is a result of the background motion being perceived as something other than the true background motion in the optic flow pattern (β_percept_ < 1) or whether it is a result of making less use of that perceived motion (β_utilize_ < 1) has not been clear until now. Here, we show that for the case of laminar translational flow and radial expansive optic flow, a misperception of the relevant motion component in the background flow (β_percept_ < 1) accounts, on average, for all of the deviation of β_total_ from unity.

Our matching results indicate that even when the appropriate velocity for subtraction is signaled very clearly—all parts of the background moving at the appropriate velocity—the *perceived* background flow is of a lower magnitude than it actually is. In other words, the participants do not match the target to the actual, very obvious, sideways velocity but instead, match it to a slower-moving version of the background. This finding has significance when considering the processing of background information during target perception tasks—a topic that has received little attention. What these results suggest is that, in lab settings, not only is target perception altered by the presence of a moving background but also the perception of the background is also altered by the presence of a target (Rock et al., 1980).

In fact, for Experiments 1 and 2, the misperception of the target can be fully accounted for, by a misperception of the background for the group. There is no evidence in the group data that β_utilize_ is different from 1, leaving the deviation of β_total_ from 1 to β_percept_'s deviation from 1. On the other hand, in Experiment 3, β_utilize_ is significantly less than 1 for the group. That is, there is strong evidence that something other than a misperception of the background is affecting target perception. Comparing the Experiment 3 stimulus to those of Experiments 1 and 2 should provide clues as to what that other factor may be.

In all three experiments, there is a global sideways motion for the background where that motion can be obtained by taking the average of the motions of all local elements. The thing that distinguishes Experiment 3 background from those of the other two experiments is that in Experiments 1 and 2, the local elements have motions that are precisely aligned with the global optic flow pattern. That pattern was translational in Experiment 1 and expansive in Experiment 2. In Experiment 3, on the other hand, the local element velocities were scattered randomly around the global velocity. This random scattering of local velocities around the mean can be considered noise.

Below we present two models that can account for our results. The first accounts for those of Experiments 1 and 2, and the second includes a modification that is needed to account for the results of Experiment 3.

### Models

**Figure 8.**
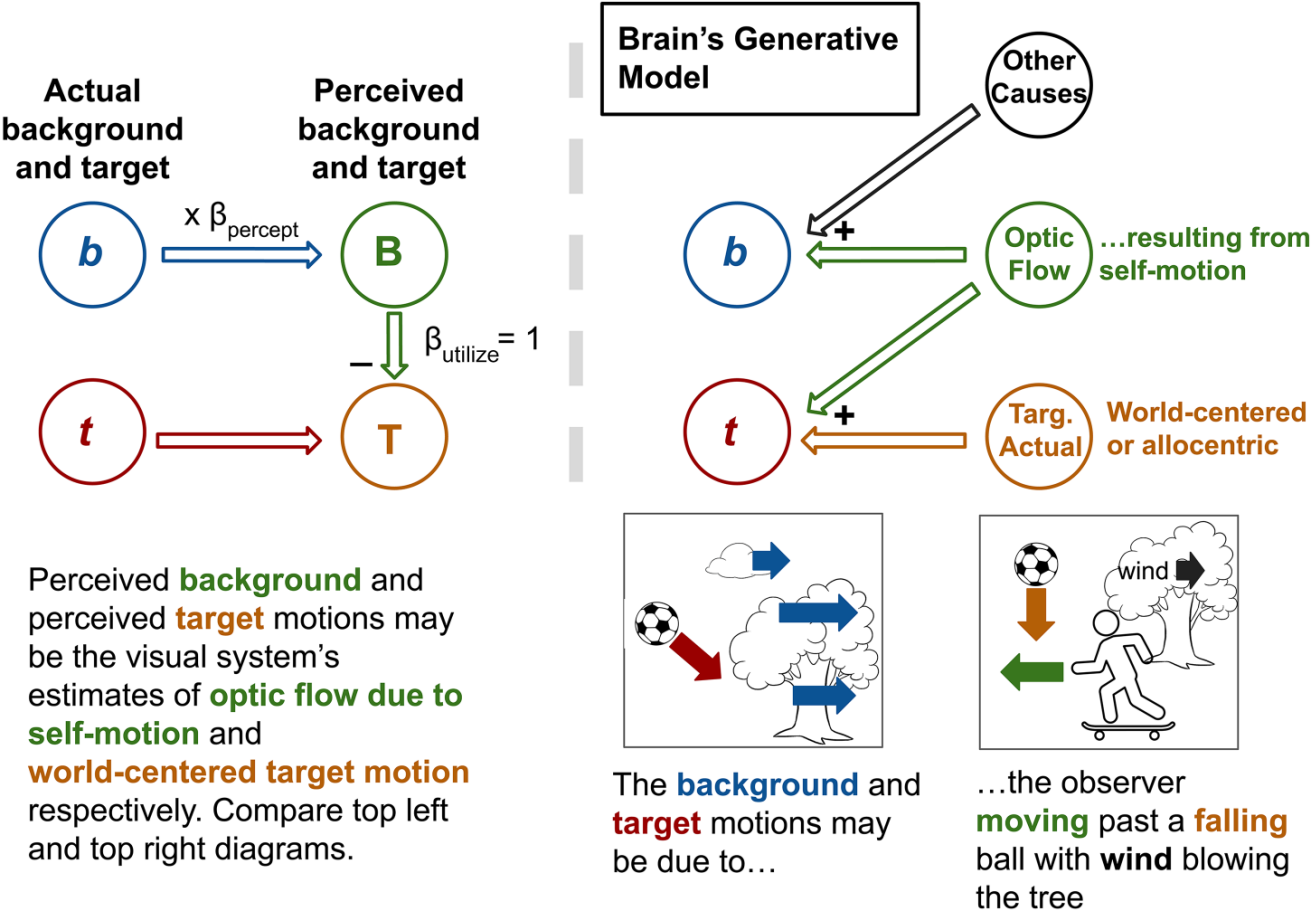
First model being tested. On the left is a depiction of the proposed process in the visual system for producing perceived target and background motions from stimulus target and background motions and on the right is a proposed internal model of the stimulus generative process that is the basis for the process depicted on the left. We test the hypothesis that perceived background motion, **B** in the top left diagram, is the visual system's estimate of background motion due to self-motion called “Optic Flow” in the top right diagram. If that is the case, following the vector addition rules depicted in the top right diagram, the only way for perceived target motion (**T** on the left equated with “Targ. Actual” on the right) to be **t** minus something-less-than-**b** is for there to be “Other Causes” assigned to the background motion in the scene, **b**. This would result in a decrease in the magnitude of **B**, and this decrease in magnitude would account for all of the something-less-than-**b**-ness, that is, β_total_ = β_percept_. If all of β_total_ is accounted for by β_percept_ then β_utilize_ = 1 as shown in the diagram on the left.

By probing perceived background motion, we propose to have decomposed β in equation (2) into two parts in an effort to understand the contributions of each part to the incompleteness of subtraction during flow-parsing in lab settings. The right panel of Figure 2 in the Introduction depicts the resulting relationship between the various quantities made available for study by this parsing. Here, we begin by presenting a model that assigns specific meanings to these quantities and predicts specific value ranges for some of these quantities. This model is depicted in Figure 8.

The model leans heavily on the Inferential/Bayesian Brain proposal (Clark, 2013; Knill & Pouget, 2004) that perceptual systems attempt to estimate the hidden causes of sensory stimuli, that is, that perceptual systems contain “generative models” of the stimuli they encounter. For a scene like that depicted on the bottom left of the right half of Figure 8, there are many possible real-world causes for the stimulus motion vectors. For the target, there are two possible causes: the actual motion of the target (relative to the world) and the scene motion at the location of the target due to the self-motion of the observer. The target motion in the stimulus is precisely a vector sum of the two. For the background motion, the possible causes are self-motion and “Other Causes” such as wind and the actual motion of components making up the background (e.g., one component of the background may be wind-blown leaves in a tree). These motions are also vector summed to produce the actual motions of the background elements in the stimulus.

Previous research and the flow-parsing hypothesis itself equate perceived target motion **T** with the actual target motion in the world, thereby equating **T** in the top left diagram with “Targ. Actual” in the top right diagram (Rushton & Warren, 2005).

It is possible that the perceived background motion **B** is the brain's estimate of background motion due to self-motion, thus equating **B** on the left with “Optic Flow” on the right. If that is the case, we would expect the following: as **t** = “Actual Targ.” (equivalent to **T**) plus “Optic Flow” (equivalent to **B**), then **T** = **t** – **B**. Comparing this with **T** = **t** – β_total_**b** (equation (2)) makes **B** = β_total_**b** which, in turn, makes β_percept_ = β_total_ and β_utilize_ = 1. That is, any incompleteness in the subtraction process is due entirely to β_percept_. This is reflected in the fact that “β_utilize_ = 1” in the top left diagram. This makes sense according to the top right diagram in Figure 8 as the only way for the quantity subtracted from **t** to be anything less than **b** is if there is an “Other Cause” assigned to the motion in **b**. Adding another cause only decreases β_percept_, not β_utilize_.

In terms of target and background perceptions, if β_total_ is completely attributable to β_percept_ then the target misperception in flow-parsing and induced motion studies is entirely due to the subtraction of a misperceived background.

The central model prediction, that β_total_ is entirely due to β_percept_, has surprising implications when one considers the way we measured β_percept_. We did so by continuously adjusting the sideways component of background motion randomly in the vicinity of the target **b**, and having participants match a target to that sideways motion. This is the continuous matching task described in Introduction. In Experiment 1, the sideways background motion was extremely clear with each element of the uniform background moving precisely at the same sideways motion as in the vicinity of the target. If participants were not able to match a target to this motion it would be surprising. But that is exactly what our model predicts; if β_total_ is less than one as expected from previous studies, then β_percept_ will be less than one by the same amount. This surprising prediction was supported by our Experiment 1 results.

Our Experiment 2 results further confirmed the predictions of our simple model. The optic flow pattern was expansive but, just as in Experiment 1, there was a global sideways background motion. This could be obtained in three ways: by using the background motion at the very center of the target, by taking the global average background velocity over the whole scene, and by parsing the global motion into its facing direction component and the orthogonal, sideways component as shown in Figure 5. All three methods give the same result but, surprisingly, the participants matched the target to a slower version of the background making β_percept_ less than 1. The average β_percept_ value for the group accounted for the group β_total_ confirming our model. For these two experiments, β_utilize_ plays no significant role in the group results, that is, β_utilize_ is equal to 1 on average.

Two notes about “causes” of stimulus motion need to be made before Experiment 3 results are discussed. The first begins with an observation: following the flow-parsing hypothesis, it only makes sense to subtract the background if an observer believes the background motion is due to self-motion. So why would the visual system assign any credence whatsoever to background motion being caused by self-motion in a lab setting where the observer is stationary? In other words, why should we expect β_total_ to be anything other than zero when a participant is not actually in motion? In this case, there will be no nonvisual cues to self-motion such as vestibular and proprioceptive cues. This question can be answered by considering two everyday experiences. The first is sitting in a moving vehicle such as a train while looking out of the window. As long as the train is not accelerating by speeding up, slowing down, or turning, the observer will not experience any self-motion cues other than a visually moving background. In such cases, the visual system correctly assigns background motion to self-motion and treats the motions of objects outside of the window accordingly. For example, a car traveling alongside the train would appear stationary relative to the observer, but it is seen correctly as moving, not stationary. The second common experience is that of being “tricked into thinking we are moving” by watching footage from a moving camera as, for example, at the cinema. Just like in the train example, a camera following a moving vehicle gives us the sense that we are moving with that vehicle and we treat objects in the scene accordingly—seemingly without hesitation. It is not unreasonable to expect that moving dots on a screen in a lab setting might cause a similar assignment of that motion to self-motion, even in the absence of nonvisual self-motion cues.

The other thing to note about causes of background motion relates to the “Other Causes” depicted in Figure 8. Returning to a lab setting with moving dots on a screen, it is clear from past studies already alluded to that the illusion of self-motion is rarely complete, that is, β_total_ rarely reaches 1 in such settings. What we propose in our model is that this is due to the partial assignment of background motion to other causes. An example of another cause might simply be “the dots are moving on a screen.” In this case, the observer's visual system (partially) “sees past the illusion” to the real cause of the background motion. The weighing up of possible causes of background motion is likely informed by other perceptual modalities such as the vestibular and proprioceptive systems.

**Figure 9.**
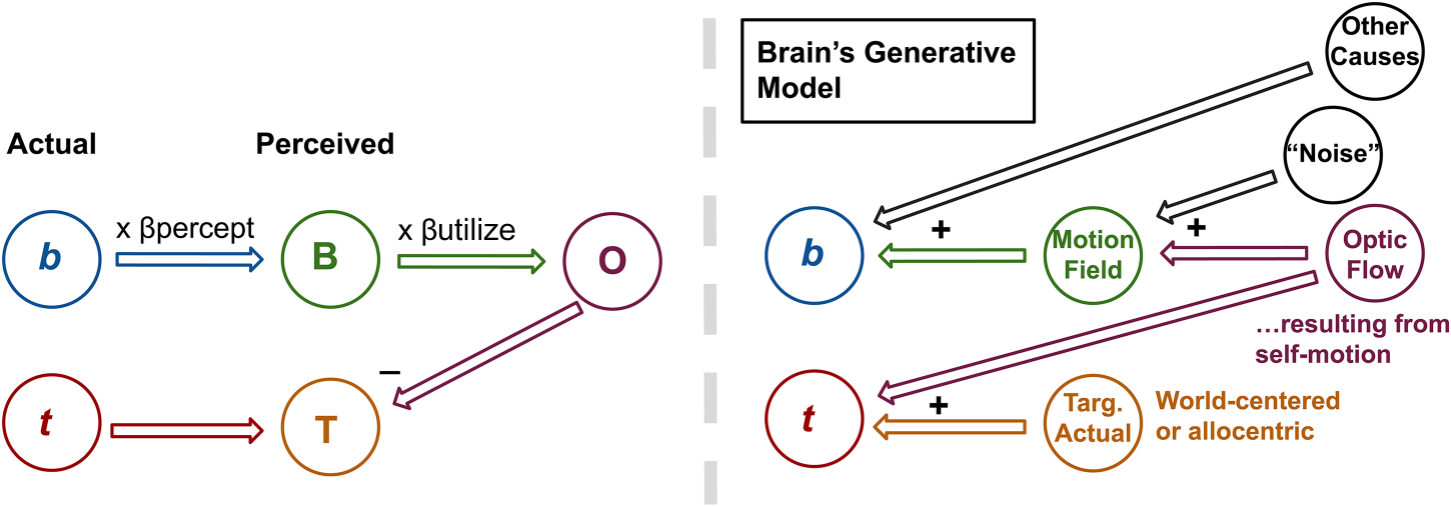
Updated model. An extra node has been included in the mechanism depicted on the left to account for the fact that β_utilize_ can be less than 1 as shown in Experiment 3. Compare with Figure 8. The visual system is still proposed to estimate optic flow due to self-motion but that is not what is perceived as the background. Instead, what is perceived is an estimate of a “Motion Field” that is intermediate between **b** and “Optic Flow due to self-motion.” This estimate of the “Motion Field” takes “Other Causes” of **b** into account and serves as input to a Bayesian-like process for estimating “Optic Flow due to self-motion.” An implication is that if **B** is “noisy” as in Experiment 3 Randomized condition, more weight will be given to prior beliefs about self-motion. If the prior belief is that self-motion is zero, then **O** will be a lower magnitude version of **B**, that is, β_utilize_ will be less than 1. The addition of the “Noise” node on the right is to indicate that when β_utilize_ is less than 1 it is because the visual system essentially assigns some of the cause of the motion in the Motion Field to “noise.” In other words, it is noise in the Motion Field that causes the estimate of Optic Flow to be less than it would be without the noise.

Our Experiment 3 results call for a more complex model than that depicted in Figure 8. That is because, in this experiment, β_utilize_ accounts for a significant portion of β_total_. A possible model update is depicted in Figure 9.

In Figure 9, the two causes of target motion in the scene are “Optic Flow” due to self-motion and the actual motion of the target, “Targ. Actual,” just as before and just as required by the vector summation processes involved in generating an image at the retina. What is different is that perceived background motion **B** is not the visual system's estimate of “Optic Flow” due to self-motion, but a “Motion Field” that is intermediate between **b** and the estimated “Optic Flow due to self-motion.” The latter is presumably represented in a higher visual area.

**Figure 10.**
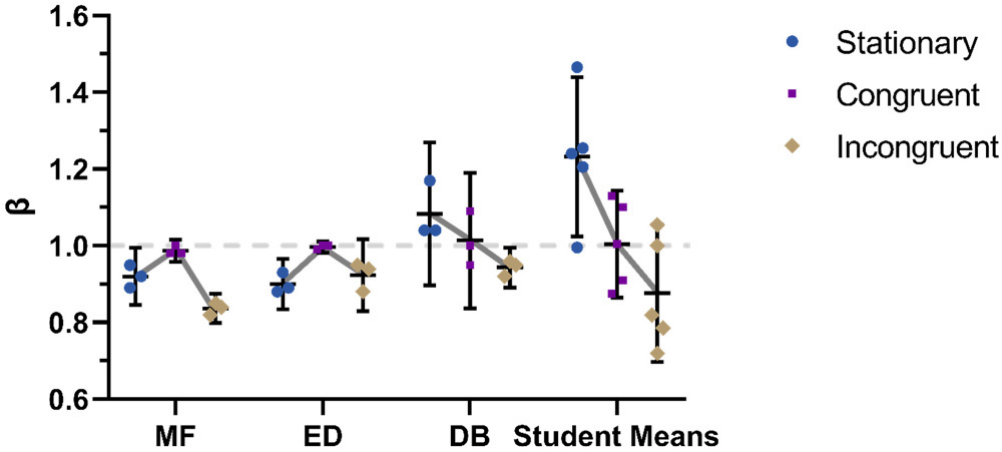
Motorized chair. Shown for MF, ED, and DB are β_total_ values for individual sessions and for each condition along with means and error bars representing 95% confidence intervals. On the right are plotted mean values based on two sessions for each of a group of 5 students (note, though, that one student only completed a single Incongruent session). In the first condition, participants were stationary, in the second the motorized chair moved congruently with the background optic flow, and in the final condition, the motion of the chair was opposite to that in the congruent condition. Some of these data have appeared previously in a different form in Falconbridge et al. (2022a).

The results of Experiment 3 randomized condition will serve to illustrate what is meant by the intermediate “Motion field.” It is possible that “Other Causes,” such as “moving patterns on a stationary screen” were partially assigned to background motion, **b**, leading to a β_percept_ less than 1 just as in the previous conditions. The resulting estimate of the “Motion Field,” **B**, was a lower-magnitude version of **b** as expected by our original model. However, the estimation of “Optic flow due to self-motion” involves a further step according to the updated model depicted in Figure 9. Holding to our Bayesian/inferential brain model of the visual system, coming to a conclusion (or posterior belief) about optic flow due to self-motion (which is a hidden cause) involves weighing up preheld conceptions (prior beliefs) about self-motion against incoming sensory data from the visual system. If we assume that preconceptions about self-motion are informed by other sensory modalities then it is likely that, for a stationary participant in a lab setting, the preconception is that self-motion is zero. But given the examples above showing that self-motion can, and often does, occur in the absence of nonvisual self-motion cues, this prior belief may only be weakly held. In other words, the prior distribution of possible self-motions may have high variance. In the case that the incoming sensory data, the “Motion Field” in this case, is very clear and has little noise associated with it, this prior may easily give way to the sensory data leading to an “Optic Flow due to self-motion” estimation that very closely matches the incoming “Motion Field” leading to a β_utilize_ close to 1. But in the case where the “Motion Field” is “noisy” as in the randomized condition in Experiment 3 where there was a great deal of variation in local motions about the mean, more weight may be given to the prior leading to an estimated “Optic Flow due to self-motion” that is somewhere between the “Motion Field” and zero, that is, a β_utilize_ less than 1.

A noncentral finding in our study noted in Experiment 3 Discussion section is that although β_utilize_ was equal to 1 on average for the group, it tended to vary among participants rather consistently across all three experiments. This is reflected in the generally ascending pattern of β_total_ values as you move from left to right in Figure 4, Figure 6, and Figure 7, while the β_percept_ values remain fairly consistent. According to our updated model, this reflects a varying tendency to hold to the preconception, furnished by nonvisual perceptual modalities, that self-motion is zero in the face of visual data suggesting nonzero self-motion; RS and MF were less “convinced” by the visual cues and DB was most convinced. According to a strict Bayesian perspective, being “less convinced” is due to either a greater variance in the incoming sensory data or lower variance in the prior.

In considering where the process depicted on the left of Figure 9 might be implemented it is important to note that β_percept_ did not vary between the expansive optic flow condition of Experiment 2 and the randomized condition in Experiment 3 where the average of the local motions was the same but the global optic flow structure was absent. This indicates that the perceived background sideways motion was indifferent to optic flow structure. At the same time, β_total_ *was* affected by the removal of the optic flow structure indicating that the intervening step between perceived background, **B**, and perceived target, **T**, which is denoted “**O**” in our diagram, takes optic flow structure into account. This is consistent with **B** being associated with area MT—an area that is capable of representing “motion fields” (Born & Bradley, 2005) and calculating average motion from dispersed local motions, but that is not sensitive to global optic flow patterns (Smith et al., 2006) (nor vestibular stimulation, Smith et al., 2012). **O** may be associated with area MST, to which MT neurons project, which has neurons tuned to global optic flow patterns (Duffy, 1998; Smith et al., 2006). Recent evidence points to **T** being associated with activity in area MT (Kim et al., 2022) making this area a possible candidate for furnishing both target and background motion perceptions. Note that there is also evidence that a specific region of MST known as area MSTl plays a role in target perception (Sasaki et al., 2019). Which brain region is associated with the perception of specific motion signals is likely flexible and dependent on the task and other factors such as training (Liu & Pack, 2017).

### The Link Between the Matching and Correction Tasks

At first glance, the matching task used to measure β_percept_ is about matching one speed to another, and the correction task used to measure β_total_ is about direction perception. How can we assume the matching task results tell us anything about perception of the background during the correction task? What ties these two tasks together?

There is a long tradition in the Induced motion literature of using a background that moves horizontally (or vertically) and, simultaneously, using a diagonally moving target whose direction is changed to find the point where it appears to move only vertically (horizontally) (see review of Induced Motion studies by Reinhardt-Rutland (1988) and more recently, Zivotofsky (2004), Farrell-Whelan et al. (2012), and Falconbridge et al. (2022b)). The logic is that the diagonally moving target will have a motion component in the direction of the background, and the direction of the target is changed until the magnitude of that component cancels out the motion induced by the moving background (the induced motion is in the opposite direction of the background motion). Following that tradition and logic, our correction task is simply about finding the horizontal component of motion in the target that matches the background component that is being subtracted by the visual system from the target (which we label β_total_**b**).

When that is understood, the link between the matching and correction paradigm becomes clear. Both are about horizontal components, but in the correction task, it is about finding the horizontal component that is perceptually subtracted from the target, and in the matching task it is about finding the perceived horizontal motion of the background.

### Can β_total_ Equal 1?

A prediction of the model depicted in Figure 9 is that (1) when the optic flow pattern is clear and (2) when that motion can be readily assigned to self-motion, β_total_ should equal 1. In a previous study conducted in our lab (Falconbridge et al., 2022a), we tested whether a β_total_ value of 1 was obtainable using physical self-motion that was consistent with the simulated self-motion in the visual stimulus. Our experiment employed a motorized chair that was under the control of the participant and a Virtual Reality (VR) headset where the visual stimulus was displayed. Participants were tested under 3 conditions: no physical self-motion (Stationary), self-motion consistent with the visually simulated self-motion (Congruent), and self-motion inconsistent with the visually simulated self-motion (Incongruent). The data are reproduced here in a little more detail than in the original study. Plotted in Figure 10 are the β_total_ values from each of three sessions for each condition for participants MF, ED, and DB. Also shown are means and 95% confidence intervals for each condition. A line at β = 1 is shown for reference. As the pattern of results for DB differed from those of MF and ED, results are included for a group of 5 naive and unpraticed students. Each data point represents a mean taken from two sessions for each student, except in the case of the Incongruent condition where one of the participants ran out of time and only did a single session.

The central finding, here, is that mean β_total_ values were close to 1 for MF, ED, DB, and the student group as a whole for the congruent condition. None of the means for this condition were significantly different to 1 (*t*[2] = 2.00, 1.00 and 0.33, *p* = 0.18, 0.42, and 0.78 for MF, ED, and DB, respectively, and *t*[4] = 0.08, *p* = 0.94 for the student group, two-tailed *t*-test). That is not the case for either of the other conditions. MF and ED were very well practiced in using the motorized chair at the time of testing, DB had very limited experience, and the students had no experience. It is telling that the results of the more practiced participants were more consistently close to 1 for this condition (see the general decrease in a spread for purple data points moving right to left in Figure 10); unfamiliarity with the chair was associated with higher levels of data variability and as familiarity increased (and variability decreased), β_total_ values were more consistently close to 1. This result is consistent with other studies showing that the addition of vestibular cues and other cues consistent with visual cues makes β_total_ approach 1 (Dokka et al., 2015; Dupin & Wexler, 2013; Fajen & Matthis, 2013; Xie et al., 2020). Our study may be the first to demonstrate a group average of exactly 1.

For the group as a whole, and for MF and DB individually, the mean β_total_ values for the Incongruent condition were significantly less than 1 (*t*[3] = 4.38, *p* = 0.02 for the group means and *t*[2] = 18.52 and 4.72, *p* = 0.003 and 0.04 for MF and DB, respectively, using a two-tailed *t*-test). This finding indicates that it was not simply the use of a motorized chair that produced β_total_ values of 1, but it was a result of the motorized chair moving *congruently* with the background motion in the display.

The results for the Stationary condition are less consistent across participants. For ED, β_total_ was not significantly different from that in the Induced Motion condition in Experiment 1 (*t*[2] = 0.83, *p* = 0.45, from two-tailed *t*-test), for MF, β_total_ was higher than in Experiment 1 (*t*[2] = 2.87, *p* = 0.045, from two-tailed *t*-test), but in both cases β_total_ was less than 1 just as in Experiment 1 (*t*[2] = 4.62 and 6.55, *p* = 0.04 and 0.02 for MF and ED, respectively, from two-tailed *t*-test). This is not surprising as the stimulus was the same, only presented via a Head Mounted Display (HMD) rather than on a computer monitor. For DB, the mean β_total_ value was greater than 1, though not significantly. For this reason, the student data were obtained. These data confirmed the suggested trend in DB's results in that the mean β_total_ value was significantly greater than 1 (*t*[4] = 3.10, *p* = 0.04, from two-tailed *t*-test). Establishing a reason for the inconsistency in the stationary condition is beyond the scope of this study. A simple explanation is that due to previous extensive use of the motorized chair, both MF and ED interpreted the visual background motion as being caused by self-rotation, whereas a lack of exposure to the chair and a lack of depth cues in the visual stimulus led DB and the students to interpret the background motion as being caused by sideways self-translation with the target being nearer to the participant than the background. The latter interpretation of the scene allows for β_total_ values greater than 1. This is discussed in more detail in the Appendix.

### Summary

The continuous approach used here has allowed for the study of flow-parsing within the context of natural perception-action loops, has allowed for engaging testing experiences for participants, and has dramatically shortened the time needed for data collection. We also showed in the General Discussion that our continuous approach allowed for the seamless integration of nonvisual self-motion cues in a previously published study by employing a rotating chair that was controlled by the actions of the participant. Our continuous approach is an attractive alternative to slower, less interactive trial-based approaches used traditionally to study this naturally rich subtraction phenomenon.

This study provides proof-of-concept for the continuous approach presented in Falconbridge et al. (Under Review), including the data analysis method. Despite the relatively low number of participants in the current study, there was a consistent pattern across participants in the substantial within-participant data sets, and the main results were statistically significant.

We introduced our study by asking why the subtraction of the background from the target tended to be incomplete in lab settings, that is, why β_total_ tends to be less than one. What some of the studies cited here, including our motorized chair experiment, demonstrate is that under more natural conditions where participants are in control of their physical motion and when that motion is congruent with the visual stimulus, β_total_ approaches 1. This lends support to the idea that the flow-parsing hypothesis is correct under natural conditions, that is, the optic flow background due to self-motion is fully subtracted, producing perceived target motions that are relative to the scene. This concept is supported by other studies not yet cited here (Fajen et al., 2013; Ilg et al., 2004; Matsumiya & Ando, 2009).

When visual self-motion cues are not consistent with nonvisual cues as in most lab settings, the subtraction tends to be incomplete. We have shown that the incompleteness of the subtraction can be entirely accounted for by a misperception of the background in the case that the local background motion elements are consistent with the overall global optic flow in the background. When they are not consistent then the degree to which perceived background is utilized in the subtraction process also plays a role.

Finally, in setting up our approach in the Introduction, an assumption was made that perceived background motion **B** was associated with an area in the visual system somewhere between the stimulus **b** and the area associated with target perception **T**. Our results support this assumption. By probing background perception in this study, we have demonstrated that we can effectively split the factor determining how much the background is subtracted from the target during target motion judgment tasks into two parts: one which occurs prior to background perception, β_percept_, and another that occurs between background perception and target perception, β_utilize_. The first appears to be affected by the visual system's assignment of background motion to causes other than self-motion, and the second by noise in the background flow signal. This study provides a base model from which more refined models may evolve as new data emerge. Our continuous approach offers an exciting means for exploring this rich phenomenon under more natural conditions.

## Supplemental Material

sj-m-1-ipe-10.1177_20416695231214439 - Supplemental material for Target motion misjudgments reflect a misperception of the background; revealed using continuous psychophysics Supplemental material, sj-m-1-ipe-10.1177_20416695231214439 for Target motion misjudgments reflect a misperception of the background; revealed using continuous psychophysics by Michael Falconbridge, Robert L. Stamps, Mark Edwards and David R. Badcock in i-Perception

sj-m-2-ipe-10.1177_20416695231214439 - Supplemental material for Target motion misjudgments reflect a misperception of the background; revealed using continuous psychophysics Supplemental material, sj-m-2-ipe-10.1177_20416695231214439 for Target motion misjudgments reflect a misperception of the background; revealed using continuous psychophysics by Michael Falconbridge, Robert L. Stamps, Mark Edwards and David R. Badcock in i-Perception

sj-m-3-ipe-10.1177_20416695231214439 - Supplemental material for Target motion misjudgments reflect a misperception of the background; revealed using continuous psychophysics Supplemental material, sj-m-3-ipe-10.1177_20416695231214439 for Target motion misjudgments reflect a misperception of the background; revealed using continuous psychophysics by Michael Falconbridge, Robert L. Stamps, Mark Edwards and David R. Badcock in i-Perception

**Figure:** Video 1: Laminar translational background flow used in Experiment 1. Here the target moves directly upwards and the background to the right. The target appears to move up and to the left. This is due to the subtraction mechanism at the heart of this study where perceived target motion is actual target motion minus some portion of background motion; **T**=**t** – β_total_**b**.

**Figure:** Video 2: Radially expansive optic flow with offset FOE. Here the FOE is to the left of the centre of the target simulating a participant fixating a target that is to the right of where they are heading (compare to Figure 5). This produces a rightward component of global motion just as in Video 2 which makes the vertically moving target appear to move up and to the left.

**Figure:** Video 3: Randomised flow field. Here, the average flow of the background is leftwards but local element velocities are “random”—pointing in seemingly random directions and moving at seemingly random speeds. See text for how this stimulus was created. The leftward average motion perceptually adds a rightward component to the vertically moving target.

**Figure:** Video 4: Background and target while MF performed the matching task.

## Appendix: The Chair Experiment

The aim of the Chair experiment was to determine whether a β_total_ value of 1 was obtainable. Recall that β_total_ is composed of β_percept_ and β_utilize_. Our model suggests that β_percept_ should approach 1 as other possible causes for the background motion in a scene are eliminated. We sought to achieve this by having self-motion be completely congruent with the background motion in the display. The medium for self-directed motion was a rotating motorized chair controllable by a handheld VR controller, and the medium for visual stimulation was an HMD.

The results of our third experiment along with our second model in Figure 9 suggest that β_utilize_ will approach 1 as the noise in the background global motion is minimized. Where there was no deviation of local motion signals around the global optic flow pattern in Experiments 1 and 2, β_utilize_ was equal to 1. When local deviations about global flow were introduced in Experiment 3, β_utilize_ values became less than 1. For this reason, a clear optic flow pattern was used in the chair experiment where each local motion element does not deviate from the global optic flow pattern. The Induced Motion background of Experiment 1 was used as it is conducive to self-rotation.

Note also that previous studies indicate that autonomy of physical motion is an important factor in making β_total_ as close to 1 as possible; compare passive movement (Dokka et al., 2015; MacNeilage et al., 2012) with active (Dupin & Wexler, 2013; Dyde & Harris, 2008; Xie et al., 2020) and note Wexler et al. (2001). To that end, the physical rotation of the participant was under their own control. As we wanted to test the effect of the congruency between self-motion and visual background motion, these two things were linked—either being congruent or not. As a result, participants kept the target moving vertically, not by controlling the target directly, but by adjusting their own motion so that it produced background motion that was appropriate for making the target appear to be moving vertically. Accordingly, opposite to Experiments 1–3, in the chair experiment the *target* motion was randomly perturbed and participants kept it perceptually moving upward by adjusting the *background*. Note however that all participants reported being unaware that they were controlling the target only indirectly. Instead, it felt as if they were in direct control of the target's motion.

In the main Congruent condition, the participant controlled the rotation of the chair using the VR controllers and the visual background moved congruently with head rotation as if the participant were rotating within a stationary world. A participant could make a target that was moving up and to the left, for example, appear to move straight upward by rotating the chair rightward, causing the background to translate more leftward.

There were two other conditions included to allow comparison of the results with those of Experiments 1–3 and to shed further light on the contribution of the chair. In the first stationary condition, the visual stimulus and the method for correcting for deviations in target velocity from vertical were the same as the congruent condition but there was no chair rotation. In the second incongruent condition, all was the same as in the congruent condition except that the chair rotated in the opposite direction to what it did in the congruent condition. A deviation of the target to the left of vertical was still fixed by moving the controller to the right, but the chair moved *leftward* in this case, not rightward. The accompanying head rotation caused the background to visually flow more leftward which is inconsistent with rotation of the participant in a stationary world.

Note that an interesting difference in the pattern of results between the three participants who took part in all four experiments led us to expand the participant pool. Five students, naive to the experimental goals and unpractised in the task, were also included in this study.

### Methods

#### Participants

MF, ED, and DB were experienced psychophysics observers. MF and DB were aware of the purposes of the study but only MF was aware of how the experimental tasks related to that purpose. Participant ED had a divergent squint and completed the experiments using an opaque eye patch over the nondominant eye. All student participants were unaware of the purpose of the experiments, and none were experienced psychophysical participants. All participants gave their informed consent to the study which had ethics approval (RA/4/1/4503) from the Human Ethics Committee at the University of Western Australia and therefore conformed to the tenets of the Declaration of Helsinki.

#### Apparatus

Stimuli were presented via an HTC Vive Pro HMD. The visual stimuli were created and updated using Unity version 2020.2.7f1 via SteamVR version 1.18.7, both running on a Gigabyte Sabre 15 laptop running a Windows 10 operating system. Chair motion (in the Congruent and Incongruent conditions) or background motion (in the Stationary condition) was controlled using a handheld HTV Vive Pro controller. The chair motor speed commands were sent by Unity to an Arduino Mega 2560 board via the Ardity (version 1) Unity plugin and Arduino software (version 1.8.12). The Arduino board relayed the commands to a Nema 34 12NM Servo Motor Hybrid Driver that controlled the stepper motor speed and direction. The motor was attached to a standard swivel office chair. See Falconbridge et al. (2022a) for further details.

#### Stimuli

All stimulus properties mimicked those in Experiment 1 as closely as possible except that the plaid patches making up the target and background appeared on a plane in a virtual environment that was 8 m from the participant. The patches and display plane were enlarged so that their size, the target and background areas and plaid speeds were the same as Experiment 1 in terms of visual angle. The display area was so far away in virtual space so as to reduce the risk of the visual system assigning background motion to the motion of a texture clearly seen to reside on a nearby display plane. The lack of stereo depth cues at 8 m in a digital world (the same image being presented to both eyes) meant that there were many possibilities for perceived distance of the background and target from the participant. The stimulus properties only indicated that both were far away.

For all three conditions, the target motion direction stepped either clockwise or anticlockwise by 1° every 0.1 s with a 50% chance of each. In all three conditions, if the target motion appeared to be anticlockwise or vertical the participant moved the controller to the right, and if clockwise or vertical then they moved the controller to the left. In all three conditions, the appropriate magnitude of movement would correct the discrepancy, but the way it did so was different for each condition. In the Stationary condition, a rightward controller movement increased the leftward speed of the background. In accordance with the subtraction mechanism at the heart of this study, the more leftward velocity of the background would be subtracted from the too-leftward velocity of the target to produce a new perceived target that was nudged to the right, that is, more vertical. In the congruent condition, the same rightward controller movement would cause the chair rotation speed to increase in the rightward direction from the point of view of the participant. The resulting more rightward rotation of the head would drive the background in the display more leftward, just as if the participant were rotating in a stationary environment where the background remains stationary in real-world coordinates. This more leftward background motion should nudge the perceived target direction to the right just as in the stationary condition. In the incongruent condition, all was exactly the same as in the congruent condition except that the chair moved in the opposite direction. A rightward movement of the controller would produce a more leftward motion in the background via the head movement of the participant, but the chair would also move leftward simulating a world where turning to the left causes the world to spin twice as fast as the chair to the left.

Training sessions involved matching the horizontal motion of the background to the motion of the target that moved only horizontally. The speed of the target stepped either rightward or leftward by 0.6 °/s every 10 s. In stationary training sessions, the background motion was controlled in the same way as it was in the (nontraining) stationary condition just described. In congruent training sessions, the background motion was controlled via chair motion just as in the congruent condition above. Participants were asked to match the background to the target as soon as a step in the target speed was noted.

#### Procedure

The procedure for MF, ED, and DB differed slightly from that of the students due to time limitations on student participation. Both groups completed sessions in the same order: stationary training sessions similar to those in Experiment 1 so that the Bayesian Participant model parameters could be set for the participant, stationary sessions, chair training sessions similar to the stationary ones but where the chair moved, congruent sessions, and incongruent sessions. The difference was that the nonstudent group had ample time to practice each type of session so that a level of competence could be achieved, and data were collected for three sessions. Each student only had 2 h so practice was minimal and there were only two data-collection sessions for each condition. If students completed more than two of any type of session, only the last two sessions were analyzed.

#### Data Analysis

Data analysis for the experimental sessions involved the same steps as for Experiments 1–3. There were two differences: the target direction was treated as the independent variable and the background sideways speed as the dependent variable during analysis and the chair background data were smoothed by removing excessively large peaks/troughs and applying a 2 s averaging filter. The filter was needed because the raw data had large discontinuities due to the fact that it was a direct function of head position and head movements tended to be jerky, especially when the chair speed suddenly changed. Analysis of training data was similarly modified.

### Discussion of Stationary Results

There are at least two possible explanations for the stationary condition β_total_ values being larger than 1 for DB and the student group. The first is that the mechanisms of the experiment led to the target being treated as a background, and the background as a target for DB and the students. We have suggested previously that target and background motions are treated equally by the visual system in lower and midlevel visual processing stages until, at a certain later stage, attention directed at the target diverts the target to an “object pathway” and the background to an “optic flow/background” pathway (Falconbridge et al., 2022b). In this experiment, participants were given direct control of the background rather than the target so that the chair could be under the control of the participant—the chair motion being linked to the background motion. This may have lead DB and the students to attend to the background rather than the target which may have shunted the target to the “background” pathway and the background to the “object” pathway essentially swapping **t** and **b** in the matching equation in Introduction. In this case, β_total_ is being applied to **t** rather than to **b** which should result in the measured β value actually being 1/β_total_.

There are at least two problems with this possibility. The most critical is that participants reported attending to the target during all sessions, not the background. It felt to them as if they were directly controlling the target so they focused on the target motion in an effort to correct for deviations from vertical. The second is that this inversion of β values from the usual less-than-one to greater-than-one only occurred for the stationary chair condition; why not for the others? The task and the mechanism for achieving the task were the same for all three conditions; the only difference was that the chair moved in the other two conditions.

A more harmonious possibility is that MF and ED interpreted the cause of the background motion differently from the others. Recall that laminar translation of the background occurs naturally under two self-motion conditions. One is rotation of the participant and the other is sideways translation (relative to gaze direction) of the participant through a stationary environment. Self-rotation leads to a uniform leftward or rightward flow field regardless of the depth of objects making up the scene. For self-translation, the speed of flow depends on the distance of objects from the participant; nearer objects will appear to move faster than farther ones (the phenomenon is commonly called “motion parallax”). It is possible that MF and ED's previous extensive experience with rotating on the chair under experiment-like conditions meant that they defaulted to the self-rotation interpretation, even when the chair was stationary. The lack of previous experience linking the visual stimulus to rotation on the chair may have led DB and the students to assume the self-translation interpretation. Only under this interpretation is it possible to get β_total_ values larger than 1 and only if the target is seen as being in front of the background. This is because a nearer target would need to move faster than the background in order to appear stationary relative to the background. As the target and background were displayed on a plane that was 8 m away in the simulated VR environment, there were no stereo cues indicating the depth of the target relative to the background so this interpretation was possible. This possible explanation of the results is harmonious with the results of the other conditions because when the chair was rotating, the β_flow_ > 1 phenomenon disappeared. In this case, participants were forced by nonvisual cues to take the self-rotation interpretation.

As our purpose was to determine whether it is possible to achieve β_total_ values of 1 using physical rotation that is congruent with the optic flow, we did not pursue the cause of the β_total_ > 1 phenomenon under the stationary condition. The reason for this phenomenon remains unknown and is a potentially interesting area of future research.
